# Global Analysis of miRNA Gene Clusters and Gene Families Reveals Dynamic and Coordinated Expression

**DOI:** 10.1155/2014/782490

**Published:** 2014-03-25

**Authors:** Li Guo, Sheng Yang, Yang Zhao, Hui Zhang, Qian Wu, Feng Chen

**Affiliations:** ^1^Department of Epidemiology and Biostatistics and Ministry of Education Key Lab for Modern Toxicology, School of Public Health, Nanjing Medical University, Nanjing 211166, China; ^2^State Key Laboratory of Reproductive Medicine, Department of Hygienic Analysis and Detection, School of Public Health, Nanjing Medical University, Nanjing 211166, China

## Abstract

To further understand the potential expression relationships of miRNAs in miRNA gene clusters and gene families, a global analysis was performed in 4 paired tumor (breast cancer) and adjacent normal tissue samples using deep sequencing datasets. The compositions of miRNA gene clusters and families are not random, and clustered and homologous miRNAs may have close relationships with overlapped miRNA species. Members in the miRNA group always had various expression levels, and even some showed larger expression divergence. Despite the dynamic expression as well as individual difference, these miRNAs always indicated consistent or similar deregulation patterns. The consistent deregulation expression may contribute to dynamic and coordinated interaction between different miRNAs in regulatory network. Further, we found that those clustered or homologous miRNAs that were also identified as sense and antisense miRNAs showed larger expression divergence. miRNA gene clusters and families indicated important biological roles, and the specific distribution and expression further enrich and ensure the flexible and robust regulatory network.

## 1. Introduction

The small non-coding RNA regulatory molecules, microRNAs (miRNAs), play an important role in multiple biological processes through negatively regulating gene expression [1]. Abnormally expressed miRNAs may contribute to various human diseases, including cancer development, and some have been identified as potential oncomiRs or tumor suppressors [2, 3]. Some miRNAs are preferentially located at fragile sites and regions and are abnormally expressed in cancer samples [4]. Those deregulated miRNAs have been widely studied as potential biomarkers, especially for circulating miRNAs in human diseases [5–7].

miRNAs in gene cluster or family may have functional relationships via coregulating or coordinately regulating biological processes [8, 9], although they have various expression levels due to complex maturation and degradation mechanisms [10–12]. These clustered miRNAs are quite popular in metazoan genomes, and they may be involved in homologous miRNA genes via duplication evolutionary histories [13–15]. Simultaneously, the phenomenon of multicopy miRNA precursors (pre-miRNAs) further complicates the distributions of miRNA gene cluster and family and also implicates the dynamic evolutionary process in the miRNA world [15, 16]. The systematic analysis based on clustered and homologous miRNAs is quite necessary to unveil the potential functional correlation and contribution in tumorigenesis.

In the present study, to further understand the potential expression and functional correlations between miRNAs, we performed a global analysis of miRNA gene clusters and families in breast cancer using small RNA deep sequencing datasets. These related miRNAs may have higher sequence similarity (homologous miRNAs) or may be expressed in a single polycistronic transcript with close physical distance on chromosome (clustered miRNAs). They have been identified as cooperative regulatory molecules via contributing to multiple biological processes. Simultaneously, they also have close phylogenetic relationships through complex evolutionary process. Based on their functional and evolutionary relationships, the expression analysis will provide information of indirect interaction between miRNAs and potential contribution in cancer development.

## 2. Materials and Methods

### 2.1. Source Data

High-throughput miRNA sequencing datasets of 4 paired tumor (breast cancer) and adjacent normal tissues (P1, P5, P6, and P7) were obtained from Guo et al. [17]. The information on miRNA gene clusters and families was obtained from the public miRBase database (Release 19.0, http://www.mirbase.org/). Abundantly expressed miRNA gene clusters and families were collected and further analyzed according to relative expression levels. To comprehensively track the expression profiles between clustered or homologous miRNAs, we collected and analyzed all the members of miRNA clusters and families if one member was abundantly expressed in a sample.

### 2.2. Expression Analysis

The expression patterns were estimated using the relative expression levels (percentage) in every miRNA gene cluster or family. Simultaneously, due to dynamic expression across different individuals, equally mixed datasets were also used to estimate the expression patterns. We analyzed the potential relationships between miRNA gene clusters and families, especially some miRNAs could be yielded by multicopy pre-miRNAs. According to abundantly expressed miRNAs, we attempted to discover the potential cross-distribution and expression patterns between clustered miRNAs and homologous miRNAs. Moreover, we also focused on those clustered miRNAs and homologous miRNAs that were identified as sense and antisense miRNAs in the specific genome locus. Further expression analysis was performed based on the 4 paired datasets and mixed datasets, respectively.

### 2.3. Gene Ontology Enrichment Analysis

Experimentally validated target mRNAs of deregulated miRNAs were obtained from the miRTarBase database [18]. For those miRNAs with less or no validated targets, target mRNAs were predicted based on “seed sequences” using the TargetScan program [19]. According to these target mRNAs of deregulated miRNA gene clusters and families, the functional enrichment analysis was performed using CapitalBio Molecule Annotation System V4.0 (MAS, http://bioinfo.capitalbio.com/mas3/).

## 3. Results

Abundantly expressed clustered and homologous miRNAs were selected to perform further analysis. Some abundantly and abnormally expressed miRNAs (such as miR-23a, miR-23b, miR-24, miR-222, and miR-29a) had been experimentally validated using real-time PCR in breast cancer samples [20]. Interestingly, we found that many miRNA gene clusters and families had close relationships or had overlapped members (Tables S1 and S2; see Supplementary Material available online at http://dx.doi.org/10.1155/2014/782490). Some miRNAs could be yielded by different pre-miRNAs, and the phenomenon of multicopy pre-miRNAs largely contributed to the complex relationships. Generally, these pre-miRNAs may be located on different chromosomes, different strands of the same chromosome (including sense and antisense strands), or different regions on the same strand. The various distributions complicated the compositions of miRNA gene clusters and families. For example, miR-221 and miR-222 were members of miR-221 gene family with higher sequence similarity, but they were also clustered on chromosome X and identified as miR-222 gene cluster. Homologous miRNA members could be located in different gene clusters through locating on different genomic regions or different chromosomes. For example, miR-23a and miR-27a were clustered on chromosome 19, while miR-23b and miR-27b were located in a cluster on chromosome 9. Simultaneously, sense and antisense miRNA genes were also involved in the gene cluster and family (Tables S1 and S2). miR-103a and miR-103b were homologous miRNA species (they were homologous members in miR-103 gene family), while their precursors were located on the sense and antisense strands of chromosomes 5 and 20, respectively (miR-103a-2 and miR-103a-1 gene clusters could be detected based on their multicopy pre-miRNAs).

**Figure 1:**
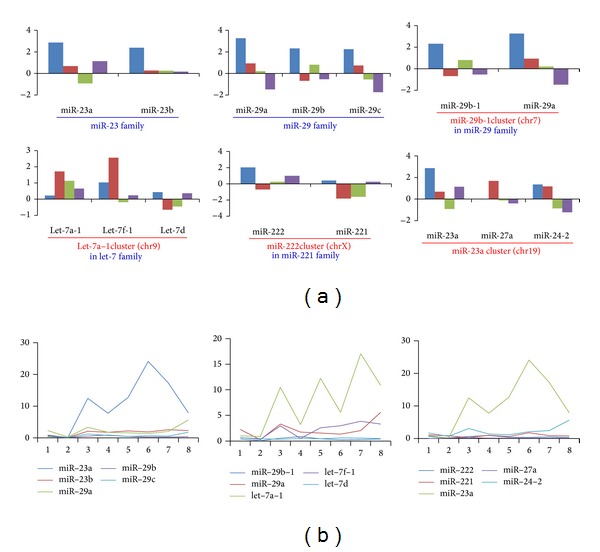
Examples of deregulation patterns of miRNA gene clusters and families (a) and their dynamic expression patterns (b). (a) Members in these miRNA gene clusters and families may be repeated. Some clusters are members of a specific gene family. The horizontal axis indicates the miRNA gene cluster or gene family; and the vertical axis indicates the fold change value (log_2_) based on each pair of tumor and adjacent normal samples. Bars in different colors (blue, red, green, and purple) indicate fold change value (log_2_) of the four pairs of tumor and adjacent normal samples, respectively; (b) dynamic expression across the 8 involved samples (P1-tumor, P1-normal, P5-tumor, P5-normal, P6-tumor, P6-normal, P7-tumor, and P7-normal). The horizontal axis indicates the 8 samples, and the vertical axis indicates the relative expression (percentage).

**Figure 2:**
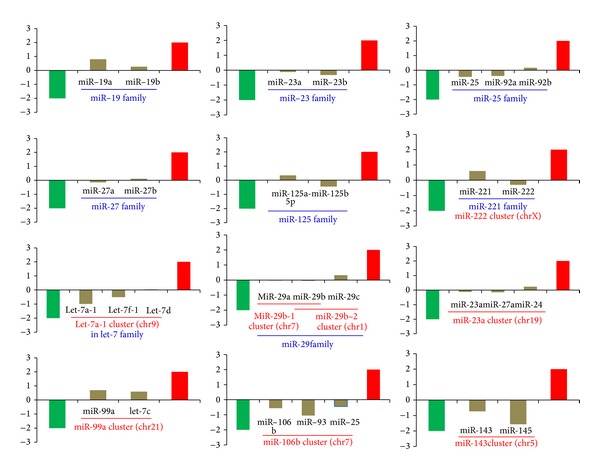
Examples of consistent or similar deregulation patterns in clustered and homologous miRNAs based on the equally mixed datasets. The horizontal axis indicates the miRNAs and the involved gene cluster and family, and the vertical axis indicates the fold change (log_2_) based on the equally mixed datasets of tumor and normal samples. The green and red bars indicate the threshold values (2 and −2).

Clustered and homologous miRNAs always showed consistent deregulation patterns in tumor samples (Figure 1(a)), although they had various expression levels (Figure 1(b)). They might show expression divergence as well as individual difference across different samples. The dynamic expression patterns in miRNA gene clusters and families were quite popular, even though they might be cotranscribed as a single polycistronic unit or had higher sequence similarity. For example, one member was abundantly expressed, while another clustered or homologous member had lower expression level (Figure 1(b)). The deregulation patterns were also influenced by the various expression levels, especially some were rarely expressed. The fold change (log_2_) showed larger divergence between different clustered or homologous miRNA species and between different individuals (Figure 1). Furthermore, we also performed the expression analysis based on the mixed datasets. Similar expression patterns could be detected (Figure 2). The divergence of fold change existed, but the difference had been largely reduced than the expression analysis based on each pair of samples (Figures 1 and 2).

**Figure 3:**
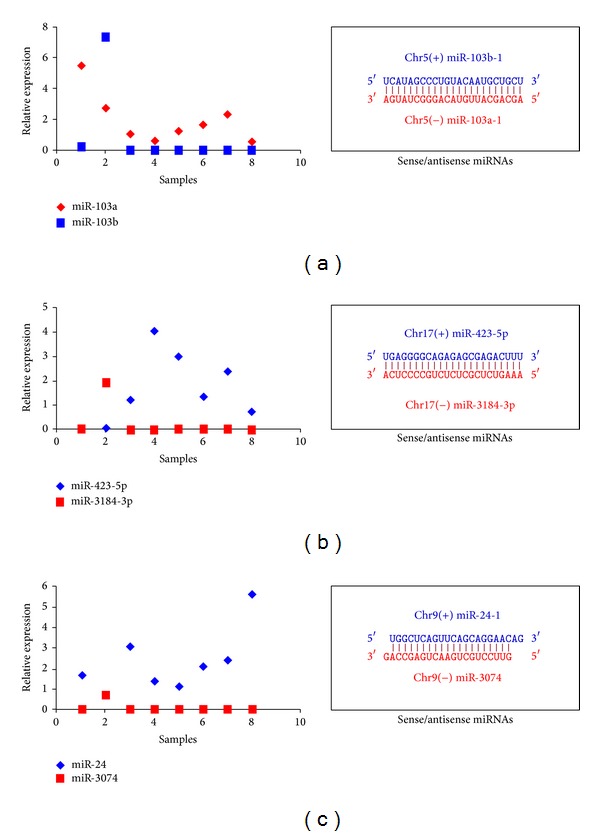
Examples of diverged expression of sense and antisense miRNAs in miRNA gene clusters and families. The detailed sense and antisense miRNA sequences are also presented on light, and they can perfectly reverse complementarily binding to each other. (a) miR-103a and miR-103b are homologous miRNAs and also clustered on chromosomes 5 and 20 as sense and antisense miRNAs (the figure only lists the sequences on chromosome 5); (b) miR-423 and miR-3184 are a pair of sense and antisense miRNAs on chromosome 17; (c) miR-24-1 and miR-3074 are sense and antisense miRNAs on chromosome 9 and also clustered in miR-23b gene cluster.

For those miRNA gene clusters and families that were involved in sense and antisense miRNAs, we also analyzed their expression patterns. As expected, they always showed larger expression divergence (or both of them were rarely expressed): if one member had abundant expression level, another would be rarely detected (Figure 3). The sense and antisense miRNAs could be perfectly reverse complementarily binding to each other, although they may also be homologous miRNA genes with higher sequence similarity.

According to the predicted target mRNAs, the common targets could be detected between clustered or homologous miRNAs (Table S3). Functional enrichment analysis of deregulated miRNA groups showed that they had versatile roles in multiple basic biological processes such as regulation of transcription and signal transduction (Table 1).

## 4. Discussion

miRNAs have been widely studied as crucial regulatory molecules, but the global expression patterns of miRNA gene clusters and families are little known. These clustered or homologous miRNAs have potential, functional, and evolutionary relationships, and they may coregulate or coordinately regulate multiple biological processes. The potential coordinated interaction complicates the coding-non-coding RNA regulatory network and enriches the miRNA-mRNA and miRNA-miRNA interactions [21, 22]. Sense and antisense miRNAs have been characterized as potential miRNA-miRNA interaction with larger expression divergence (Figure 3). Recent studies have shown that these endogenous complementary miRNAs can restrict the transcription or maturation process of one another [23–27]. The perfectly reverse binding suggests that miRNA-miRNA interaction may be a potential regulatory method in the miRNA world [21]. Further, the compositions of gene clusters and families are not random and independent, and the phenomenon of multicopy pre-miRNAs further complicates the distributions of miRNAs [28]. Clustered and homologous miRNAs always have close relationships with overlapped members (Tables S1 and S2). The interesting distributions and relationships may be mainly derived from the complex duplication history that may adapt to the functional and evolutionary pressures [13–15, 29].

Although clustered and homologous miRNA members are involved in various and inconsistent enrichment levels via maturation and degradation mechanisms, they are prone to present consistent or similar deregulation patterns in tumor samples (Figures 1 and 3). Across different samples, miRNAs may show the larger expression divergence. The reason may be partly derived from the deep sequencing datasets with higher sensitivity and potential divergence during sequencing and sample preparation. On the other hand, the individual difference also leads to the expression divergence, especially for these patients may be involved in different degrees or stages of breast cancer, although they are clinically characterized as primary breast cancer. Multiple factors may contribute to occurrence and development of breast cancer, and different samples may be prone to detect slightly inconsistent miRNA expression profiles. The dynamic expression patterns may contribute to the robust regulatory network and adapt to specific intracellular environment. Indeed, these miRNA gene clusters and families have important roles in multiple biological processes (Table 1). The consistent deregulation patterns contribute to their potential coordinated interaction, although they indicate various expression levels.

Furthermore, other factors also contribute to the expression divergence in miRNA gene clusters and families. Firstly, the phenomenon of cross-mapping or multiple mapping contributes to the relative expression levels [23, 30], especially between those homologous miRNAs. The same sequencing fragments can be mapped to different pre-miRNA sequences, and any arbitrary selection will influence the final expression analysis. Secondly, multiple pre-miRNAs have been identified that can yield the same miRNAs. However, it is hard to infer the genuine origin. These multiple pre-miRNAs are always located on different chromosomes or different strands on the same chromosomes. In the typical analysis, we always analyze the mature miRNAs and rarely consider their real origins. The default analysis would influence the expression patterns of members in miRNA gene clusters. Clustered miRNAs are characterized based on the location distributions of miRNA genes, but mature miRNAs are used to estimate the final expression levels. The arbitrary and default selection may lead to the imprecise expression analysis. Finally, an miRNA locus can yield many sequences with various 5′ and/or 3′ ends due to imprecise cleavage of Drosha and Dicer [31–33]. These multiple miRNA variants, also termed isomiRs, largely enrich the miRNA study and coding-non-coding RNA regulatory network as physical miRNA isoforms. These multiple isomiRs also influence the expression estimation, especially expression analysis based on the most abundant isomiR, the canonical miRNA, or sum of all isomiRs, respectively. Simultaneously, these various sequences also contribute to the phenomenon of cross-mapping between different miRNAs [23]. In the present study, the expression analysis at the miRNA level (based on the sum of all isomiRs) is not comprehensive. Collectively, expression divergence between miRNAs is more complexity* in vivo*, which may contribute to the dynamic regulatory network.

Taken together, although various expression levels can be detected, consistent or similar deregulation patterns are always found between clustered or homologous miRNAs. The expression patterns provide an opportunity to coregulate or coordinately regulate biological processes. Therefore, the dynamic and coordinated expression may have important biological roles, which should be derived from the functional and evolutionary pressures. As flexible regulatory molecules, multiple miRNAs can negatively regulate biological pathways based on potential coordinated interaction (e.g., based on miRNA gene clusters and families). Further study should be performed that clustered and/or homologous miRNAs would be potential biomarkers to study the mechanisms in tumorigenesis.

## Supplementary Material

Table S1 listed abundantly expressed miRNA gene clusters, Table S2 listed abundantly expressed miRNA gene families, and Table S3 indicated that homologous miRNAs or cluster miRNAs can regulate the common target mRNAs

## Figures and Tables

**Table 1:** Enriched GO terms based on experimentally validated target mRNAs in Figure 1.

GO term	Count	*P* value
GO:0006355 regulation of transcription, DNA-dependent	24	5.28*E* − 26
GO:0006350 transcription	18	7.48*E* − 17
GO:0007165 signal transduction	18	2.37*E* − 14
GO:0007275 development	15	1.82*E* − 13
GO:0006508 proteolysis	14	3.32*E* − 17
GO:0045944 positive regulation of transcription from RNA polymerase II promoter	12	1.05*E* − 21
GO:0007155 cell adhesion	10	1.67*E* − 12
GO:0006915 apoptosis	10	5.62*E* − 12
GO:0008285 negative regulation of cell proliferation	9	7.05*E* − 15
GO:0006468 protein amino acid phosphorylation	9	2.03*E* − 11
GO:0006917 induction of apoptosis	8	9.59*E* − 14
GO:0042981 regulation of apoptosis	8	2.75*E* − 10

Here, we only list important GO terms that involved at least 8 target mRNAs of differentially expressed miRNAs. Count indicates involved number of target mRNAs; *P* value indicates enrichment *P* value.
